# Solubility of *Ketoconazole* (antifungal drug) in SC-CO_2_ for binary and ternary systems: measurements and empirical correlations

**DOI:** 10.1038/s41598-021-87243-6

**Published:** 2021-04-06

**Authors:** Gholamhossein Sodeifian, Seyed Ali Sajadian, Fariba Razmimanesh, Seyed Mojtaba Hazaveie

**Affiliations:** 1 Department of Chemical Engineering, Faculty of Engineering, University of Kashan, 87317-53153 Kashan, Iran; 2 Laboratory of Supercritical Fluids and Nanotechnology, University of Kashan, 87317-53153 Kashan, Iran; 3 Modeling and Simulation Centre, Faculty of Engineering, University of Kashan, 87317-53153 Kashan, Iran; 4 South Zagros Oil and Gas Production, National Iranian Oil Company, 7135717991 Shiraz, Iran

**Keywords:** Chemical engineering, Green chemistry

## Abstract

One of the main steps in choosing the drug nanoparticle production processes by supercritical carbon dioxide (SC-CO_2_) is determining the solubility of the solid solute. For this purpose, the solubility of Ketoconazole (KTZ) in the SC-CO_2_, binary system, as well as in the SC-CO_2_-menthol (cosolvent), ternary system, was measured at 308–338 K and 12–30 MPa using the static analysis method. The KTZ solubility in the SC-CO_2_ ranged between 0.20 × 10^–6^ and 8.02 × 10^–5^, while drug solubility in the SC-CO_2_ with cosolvent varied from 1.2 × 10^–5^ to 1.96 × 10^–4^. This difference indicated the significant effect of menthol cosolvent on KTZ solubility in the SC-CO_2_. Moreover, KTZ solubilities in the two systems were correlated by several empirical and semiempirical models. Among them, Sodeifian et al*.,* Bian et al., MST, and Bartle et al. models can more accurately correlate experimental data for the binary system than other used models. Also, the Sodeifian and Sajadian model well fitted the solubility data of the ternary system with *AARD%* = 6.45, *R*_*adj*_ = 0.995.

## Introduction

Serious fungal infections can increase due to the development of the human immunodeficiency virus (HIV), anti-cancer chemotherapy, and/or the greater utilization of the immuno-suppressive treatments in transplanting the organs^1^. Ketoconazole (KTZ) is mainly applied as a synthetic imidazole antifungal drug to treat fungal infections in different forms (oral tablets, topical creams, and gels). It has been applied in immunocompromised patients and advanced prostatic carcinoma^1,2^. KTZ has very low solubility (17 µg/ml) in the water and higher penetrability; therefore, it has been considered as a class II drug in the biopharmaceutics classification system (BCS). Improving the solubility of pharmaceutical compounds is a challenging subject as it can significantly reduce the oral bioavailability and thus the therapeutic efficacy of drugs^2^.

Reducing particles' size and hence increasing the available surface area can enhance the solubility and bioavailability of pharmaceutical compounds with lower water solubility. Therefore, researchers have applied different processes (such as high-pressure homogenization, evaporation, milling, and sublimation) to reduce particle size in the pharmaceutical industry. Meanwhile, supercritical fluid (SCF) technology has received much attention in medicine to decrease the size of the particles, hence, increasing their dissolution rate and bioactivity. According to studies conducted in this field, the application of supercritical solution methods in particle formation has rapidly expanded. The solubility of drugs in SCFs should be experimentally measured for designing pharmaceutical processes^3^.

Numerous investigators have demonstrated that solute solubility in the SCFs can be substantially improved with the addition of cosolvents (polar or non-polar)^4–10^. Gurdial et al*.*^4^, measured the solubility of o- and m-hydroxy-benzoic acid in acetone-SC-CO_2_ and methanol-SC-CO_2_ binary mixtures at the temperature range of 318–328 K and the pressure range of 90–200 bar using a continual flow apparatus. Their results indicated that the addition of little amounts of the cosolvents to SC-CO_2_ largely enhanced o- and m-hydroxy-benzoic acid solubility. Huang et al*.*^11^ evaluated the equilibrium mole fraction of aspirin in SC-CO_2_ with and without acetone cosolvent. Their results showed that acetone cosolvent could cause a fivefold increase in aspirin solubility. Also, Koga et al*.*^9^ investigated influences of cosolvents (octane & ethanol) on the solubility of fatty acids, stearic acid, and stearyl alcohol in SC-CO_2_ using a flow-type apparatus and showed the higher effectiveness of ethanol on the solubilities of fatty acids than octane. Hosseini et al*.*^12^ determined the solubility of clozapine and lamotrigine in SC-CO_2_ with menthol cosolvent at the temperature range of 313–323 K and the pressure range of 123–337 bar. The applied cosolvent enhanced the solubility of both solutes in SC-CO_2_. Consequently, the solubility of numerous compounds has been experimentally determined in the SCFs^13–20^. However, the measurement of the solubility of drugs in the SCFs under diverse pressure and temperature conditions is costly and laborious^21^. In this regard, thermodynamic models have been developed to decrease the number of required experimental measurements. Compared to the other SCFs, SC-CO_2_ has been widely employed in SCF processes due to its special thermodynamic and heat transfer properties. Additionally, CO_2_ is non-toxic, non-flammable, cost-effective, abundant at high levels of purity, and environmentally-friendly with comparatively lower critical pressures and temperatures (7.38 MPa & 304.1 K)^22–27^. Several models have been applied to correlate and predict solids solubility at supercritical conditions, among which empirical and semiempirical methods^28–34^, equations of state (EoS) (including cubic and non-cubic models)^35–43^, expanded liquid model^44^, intelligent computational techniques (e.g. artificial neural networks (ANN) and least square support vector machine (LS-SVM) networks), and combination of grey wolf optimizer and support vector machines (GWO-SVM) networks^27,45^ can be mentioned. Some characteristics such as acentric factor, and molar volume, as well as the solid vapor pressures, are essential for the calculations of EoS based models. These parameters are, however, unavailable and thus must be estimated by the group contribution techniques leading to attenuated accuracy. To overcome this drawback, several researchers have applied different empirical and semiempirical models to correlate the solubility data^46–57^.

In this study, a static analysis procedure was employed to determine the solubility of KTZ in SC-CO_2_ at different temperature and pressure conditions with and without cosolvent. The solubility of KTZ in the SC-CO_2_ with cosolvent has not been experimentally measured so far. Moreover, drug solubility in the SC-CO_2_ (i.e., the binary system) was correlated by ten semi-empirical models, including Chrastil^28^, Sparks et al.^34^, Bian et al.^41^, Bartle et al.^51^, MST^32^, Kumar-Johnston^29^, Jouyban et al.^33^, as well as Sodeifian et al.^14^, models*.* Also, MST^32^, González et al.^52^, Soltani-Mazloumi^49^, and Sodeifian-Sajadian^58^ models were applied to fit the solubility data of KTZ in the ternary system. Finally, the ability of different models was investigated in terms of three statistical measures: *AARD,* %, and *R*_*adj*_.

## Experimental

### Materials

In this work, Fadak Company (Kashan: Iran) provided carbon dioxide (CAS Number 124-38-9) with the purity of 99.99%. KTZ with the purity of 99% (CAS Number 65277-42-1) was provided by Arasto Pharmaceutical Company (Tehran, Iran). The above materials were applied with no additional treatment. Also, menthol with the purity (Ph Eur) of 99.0% (CAS Number 2216-51-5) and methanol (GC) at the purity level of 99% (CAS Number 67-56-1) were purchased from Merck (German). Tables 1 presents the structures and physicochemical features of KTZ.

**Table 1:** The utilized solute structure and the respective physico-chemical features (M_w_: Molecular weight, T_m_: melting point, λ_max_: λ with maximum absorbance).

Compound	Formula	Structure	M_W_ (g/mol)	CAS Number	T_m_ (K)	λ_max_(nm)
Ketoconazole	C_26_H_28_Cl_2_N_4_O_4_		531	65,277–42-1	423 ± 2	220
L-Menthol	C_10_H_20_O		156.26	2216–51-5	314–317	489
Carbon dioxide	CO_2_		44.01	124–38-9	–	–

### Experimental apparetus

**Figure 1:**
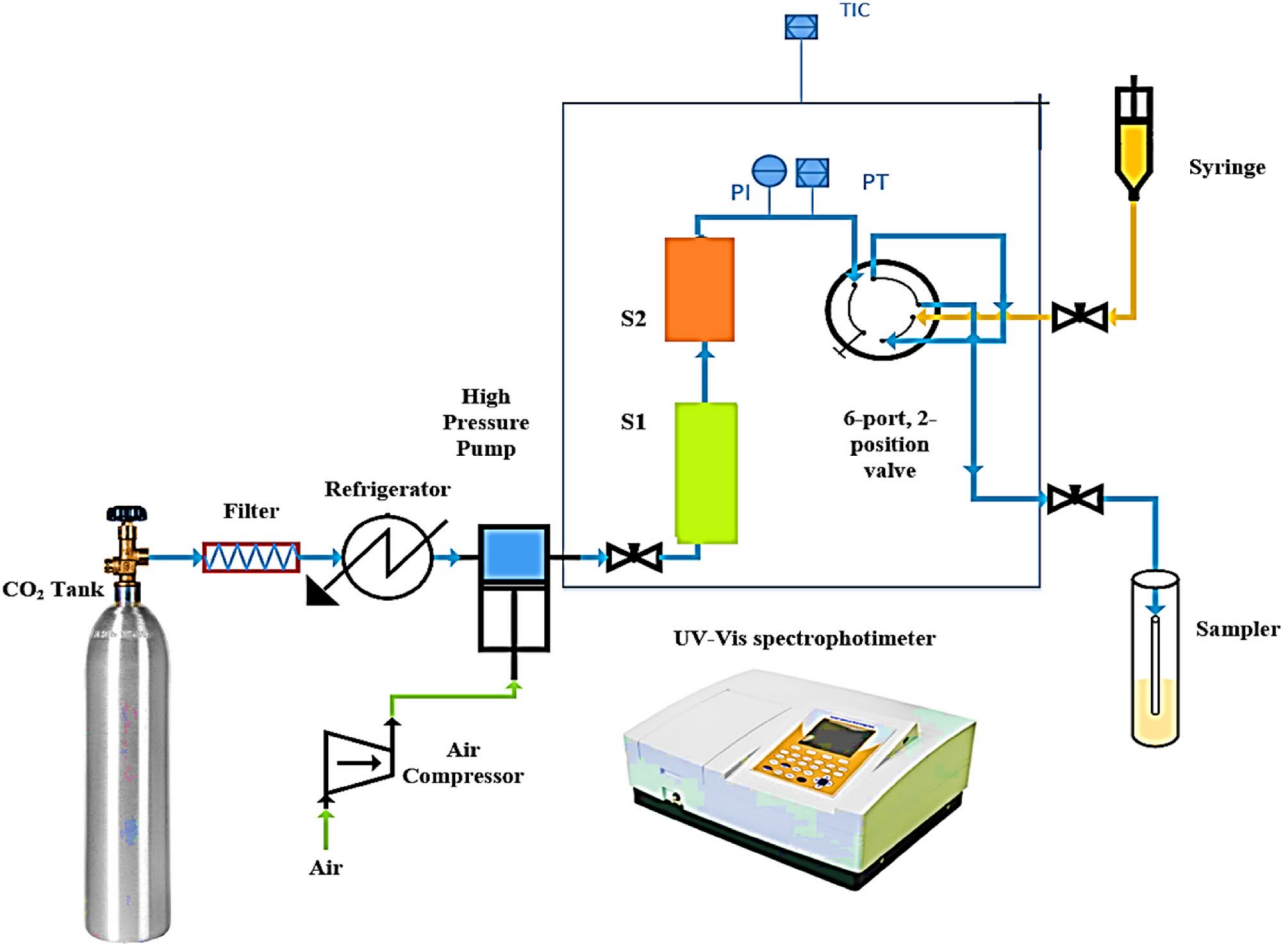
A schema of the utilized setup to measure solubility.

Figure 1 shows the laboratory setup used for determining solubility data of KTZ in SC-CO_2_ with/without cosolvent (static method). The experimental setup was completely explained in our previous paper^58^. It includes a carbon dioxide tank, filter, refrigerator unit, reciprocating pump equipped with air compressor for supplying driving force, solubility cells, pressure gauge, digital pressure transmitter, digital thermometer, oven, microliter valve, sample collector, flow meter, 1/8" piping, and connections. Pressure quantities were recorded at the accuracy of ± 0.1 MPa using both the pressure gauge (WIKA, Germany, Code EN 837-1) and pressure transmitter. To maintain the experimental temperature, the equilibrium cell was located in a precise oven (Froilabo Model, AE-60, France), which could retain the temperature within ± 0.1 K.

The amount of menthol and drug in saturator cell 1 (S1) and saturator cell 2 (S2) were 5 and 2 g, respectively. A magnetic stirrer (100 rpm) was applied to accelerate the equilibration and improve saturation of the particles in cells. The sintered filter was put on the top of the column to prevent the escape of menthol particles (as either powder or liquid droplets). In this research, the equilibrium time was considered 60 min (as determined by preliminary experiments). At the end of static time, 600 ± 0.6% µL of the saturated SC-CO_2_ was depressurized into the collection vial containing 5 ml methanol. Eventually, the loop was washed with the solvent collected in the collection vial, and the final volume of the solution was adjusted to 5 mL ± 0.6%. It should be noted that the experiments were carried out in triplicates. Consequently, the solubility of KTZ was determined by measuring the absorbance at ${\lambda }_{max}$, 220 nm (at which menthol wavelength is transparent) on the UNICO-4802 UV–Vis spectro-photometer with 1-cm pass length quartz cells. Finally, the calibration curve (with regression coefficient 0.996) was applied to obtain the medicine concentrations in the collection vial.

As presented in Tables 2 and 3, solubilities of KTZ (in the equilibrium mole fraction of the solute (y) and the grams of solute (S) per liter of SC-CO_2_ with/without cosolvent) were evaluated at the pressure range of 12–30 MPa and temperature range of 308–338 K. Finally, Span–Wagner equation was used to obtain the CO_2_ density^59^.

**Table 2:** The experimental data of KTZ solubility in SC-CO_2_ based on distinct conditions (The experimental standard deviation and the experimental standard deviation of the mean (SD) were obtained by $S({y_k}) = \sqrt {\frac{{\sum\limits_{j = 1}^n {{{({y_j} - \bar y)}^2}} }}{n - 1}}$ and $SD(\bar y) = \frac{{S({y_k})}}{\sqrt n }$ respectively.

Temperature^a^ (K)	Pressure^a^ (MPa)	Density^b^ (kg/m^3^)				Binary	
			*y*_*2*_ × 10^5^ (Mole Fraction )	Standard deviation of the mean, SD(ȳ) × (10^5^)		Expanded uncertainty of mole fraction (10^5^ U)	*S* (Solubility (g/l))
308	12	768.42	0.17	0.001		0.008	0.016
	15	816.06	0.34	0.003		0.016	0.034
	18	848.87	0.44	0.017		0.039	0.045
	21	874.4	0.62	0.017		0.044	0.066
	24	895.54	0.80	0.034		0.077	0.087
	27	913.69	0.94	0.017		0.054	0.104
	30	929.68	1.09	0.051		0.114	0.122
318	12	659.73	0.07	0.003		0.007	0.006
	15	743.17	0.32	0.010		0.027	0.036
	18	790.18	0.85	0.034		0.079	0.081
	21	823.7	1.31	0.035		0.090	0.130
	24	850.1	1.68	0.069		0.157	0.173
	27	872.04	2.11	0.035		0.115	0.222
	30	890.92	2.59	0.086		0.207	0.279
328	12	506.85	0.04	0.001		0.003	0.003
	15	654.94	0.30	0.002		0.015	0.026
	18	724.13	0.98	0.035		0.082	0.086
	21	768.74	1.82	0.052		0.131	0.169
	24	801.92	2.76	0.050		0.160	0.267
	27	828.51	4.02	0.087		0.247	0.402
	30	850.83	4.81	0.068		0.252	0.494
338	12	384.17	0.02	0.0006		0.001	0.001
	15	555.23	0.22	0.010		0.023	0.015
	18	651.18	0.90	0.017		0.056	0.077
	21	709.69	2.29	0.052		0.145	0.196
	24	751.17	4.2	0.032		0.198	0.381
	27	783.29	6.02	0.085		0.316	0.569
	30	809.58	8.02	0.121		0.427	0.784

*n* is the number of times each experimental data was measured (n = 3, in this work). Expanded uncertainty is U = k* u_*combined*_ and the relative combined standard uncertainty is defined as *u*_*combined*_* / y* = $\sqrt {\sum\limits_{i = 1}^N {{{({P_i}{\kern 1pt} u({x_i})/{x_i})}^2}} }$ *in which u(x*_*i*_*)/x*_*i*_ is the relative standard uncertainty of each input estimate (*x*_*i*_) and *P*_*i*_ is known positive or negative number having negligible uncertainties. *y*_*2*_ and *S* are mole fraction of solute in binary system and solubility of solute in SC-CO_2_, respectively.

^a^Standard uncertainty u are u(T) = 0.1 K; u(p) = 1 bar. Also, the relative standard uncertainties are obtained below 0.05 for mole fractions and solubilities. The value of the coverage factor k = 2 was chosen on the basis of the level of confidence of approximately 95 percent.

^b^Data from the Span–Wagner equation of state^62^.

**Table 3:** The experimental data of KTZ solubility in SC-CO_2_ – menthol based on distinct conditions.

Temperature^a^ (K)	Pressure^a^ (MPa)	Menthol	Ternary			
		y_3_ × 10^3^	y′_2_ × 10^4^ (Mole Fraction )	Standard deviation of the mean, SD ($\bar{y^\prime}$) × 10^4^	Expanded uncertainty of mole fraction (10^4^ U)	E (cosolvent effect)
308	12	16.40	0.27	0.003	0.014	16.1
	15	17.32	0.38	0.001	0.017	9.7
	18	18.69	0.43	0.003	0.020	9.4
	21	19.43	0.46	0.005	0.022	9.7
	24	20.43	0.54	0.003	0.024	6.8
	27	22.17	0.59	0.002	0.026	5.3
	30	23.63	0.62	0.008	0.032	4.9
318	12	14.70	0.21	0.001	0.010	30.6
	15	16.32	0.39	0.003	0.019	12.3
	18	17.34	0.53	0.008	0.029	11.1
	21	19.42	0.69	0.003	0.031	7.7
	24	21.09	0.87	0.003	0.039	5.2
	27	24.34	1.02	0.007	0.047	4.0
	30	26.36	1.11	0.009	0.051	3.3
328	12	12.36	0.18	0.005	0.013	45.9
	15	15.84	0.41	0.006	0.023	16.6
	18	18.45	0.63	0.005	0.030	11.5
	21	19.94	0.90	0.008	0.043	5.9
	24	22.34	1.12	0.004	0.050	3.9
	27	26.34	1.31	0.009	0.060	3.5
	30	29.70	1.65	0.017	0.080	2.9
338	12	12.09	0.12	0.003	0.008	61.2
	15	16.12	0.45	0.001	0.020	34.4
	18	19.18	0.76	0.003	0.034	11.6
	21	25.33	1.01	0.015	0.054	5.5
	24	24.59	1.34	0.020	0.072	3.7
	27	30.82	1.70	0.017	0.082	2.8
	30	32.87	1.96	0.025	0.100	2.4

*y*_*3*_*, y*^*'*^_*2*_ and e are mole fraction of menthol, mole fraction of solute in ternary system and cosolvent effect, respectively. The experimental standard deviation of the mean (SD) were obtained by $SD(\bar y) = \frac{{S({y_k})}}{\sqrt n }$. *n* is the number of times each experimental data was measured (n = 3, in this work). Expanded uncertainty is U = k* u_*combined*_ and the relative combined standard uncertainty is defined as *u*_*combined*_* / y* = $\sqrt {\sum\limits_{i = 1}^N {{{({P_i}{\kern 1pt} u({x_i})/{x_i})}^2}} }$ *in which u(x*_*i*_*)/x*_*i*_ is the relative standard uncertainty of each input estimate (*x*_*i*_) and *P*_*i*_ is known positive or negative number having negligible uncertainties.

^a^Standard uncertainty u are u(T) = 0.1 K; u(p) = 1 bar. Also, the relative standard uncertainties are obtained below 0.05 for mole fractions and solubilities. The value of the coverage factor k = 2 was chosen on the basis of the level of confidence of approximately 95 percent.

^b^Data from the Span–Wagner equation of state^62^.

## Results and discussion

### Binary system

In our previous study, the reliability of the solubility setup was evaluated by determining the solubility of naphthalene and alpha-tocopherol in SC-CO_2_ at different pressures and temperatures and comparing them with the corresponding data in the literature^60^. In general, the authors systematically check and calibrate the device before testing naphthalene and alpha-tocopherol solubilities in SC-CO_2_.

It should be mentioned that the mole fraction and solubility (S(g/L)) of KTZ in SC-CO_2_ were measured at different temperature and pressure conditions (Table 2). Each experimental data was measured in triplicate to enhance the data reliability. The relative standard uncertainty of the solubility data was below 0.05. The relative standard uncertainty (*U*_*s*_) can be calculated by the following equation:

1 $${U}_{s}=\frac{S({y}_{k})}{\stackrel{-}{y}}$$

2 $$ S({y_k}) = \sqrt {\frac{{\sum\limits_{j = 1}^n {{{({y_j} - \bar y)}^2}} }}{n - 1}}  $$

where $S\left({y}_{k}\right)$ and n are the experimental standard deviation and the number of measurements of each experimental data (n = 3, in this work), respectively.

y and S (g/ L) values respectively ranged between 0.20 × 10^–6^ and 8.02 × 10^–4^, and 0.001 and 0.784. Finally, the greatest and least values of KTZ solubility were observed at (338 K, 30 MPa) and (338 K, 12 MPa), respectively.

**Figure 2:**
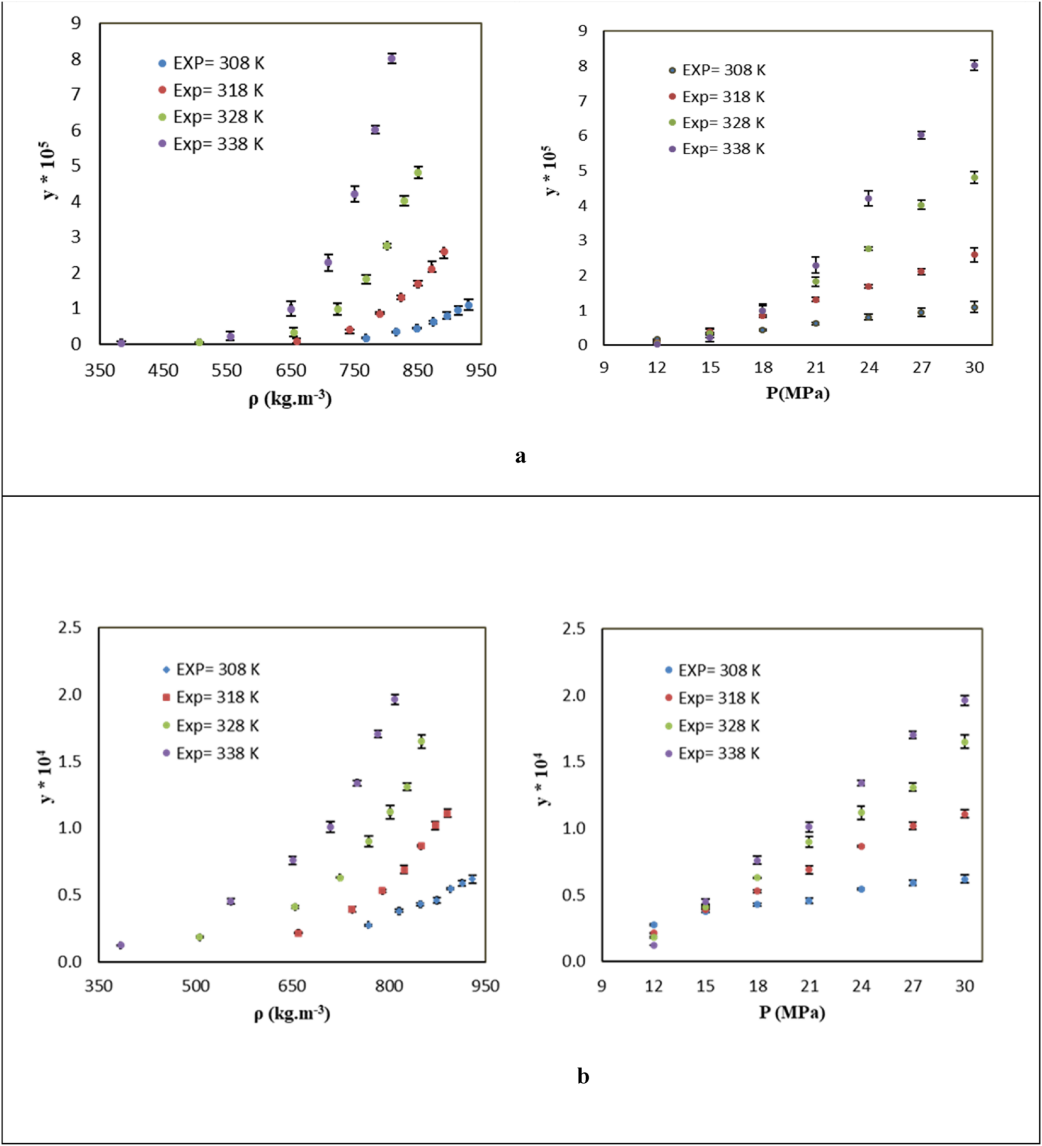
The KTZ solubility in (**a**) binary system and (**b**) ternary system.

Figure 2a shows an increase in the solubility of KTZ with pressure increment at each isotherm. An enhancement in the density also increased the solubility at the elevated pressures. Generally, SC-CO_2_ density and solute vapor pressure are the two key factors contributing to the solubility of the solute in SC-CO_2_. The solubility showed an ascending trend with increasing density and solute vapor pressure. At pressures below the crossover region, where the influence of increased solvent density on the solute solubility dominates over decreased solute vapor pressure, the solid solute exhibited higher solubility at lower temperatures rather than higher ones. At the top of the crossover region, when temperature increased, the solubility incremented more rapidly with pressure enhancement, which might be due to the competing effects of the reduction of SC-CO_2_ density and the increase of solute vapor pressure.

Figure 2a presents a pressure range of 19–20 MPa that was considered as the crossover pressure area for KTZ in the binary system. In general, several studies demonstrated that the solute vapor pressure and SC-CO_2_ density are the major parameters below and top of the crossover area^26,60–63^. Yamini and Moradi^1^ measured KTZ solubility in SC-CO_2_ at 12.2–35.5 MPa and 308–348 K considering the absorbance at ${\lambda }_{max}$ (220 nm). In the present work, the mole fraction of KTZ dissolved in SC-CO_2_ (in pressure and temperature spans of 12–30 MPa and 308–338 K) was 0.20 × 10^–6^ and 8.02 × 10^–5^. Their solubility data at this condition ranged from 0.7 × 10^–6^ to 8.16 × 10^–5^. The mean standard deviation between their experimental data and the present work was 2%. The effects of temperature and pressure on the solubility were the same for both works.

### Ternary systems

Figure 2b and Table 3 report KTZ solubility in SC-CO_2_ with cosolvent (menthol) under different pressures and temperatures. Accordingly, solubility based on the solute mole fraction (y) ranged from 1.2 × 10^–5^ to 1.96 × 10^–4^. Each experimental data was measured three times to enhance the reliability of the solubility data. Figure 2b presents an increase in KTZ solubility with the pressure increment at all isotherms. The increase of density with rising the pressure led to the more powerful solvation ability of SC-CO_2_ and thus enhanced the solid solubility. The largest increment in solubility with rising pressure was observed at the highest temperature, which can be assigned to the impacts of the temperatures and pressures on the solvent density and pressure of the solute vapor^10^. As stated previously, temperature influences the solvating power by two challenging factors: the solvent density and pressure of the solute vapor. Therefore, an increment in temperature will decrease the solubility below the crossover pressure area and also increased the solubility above the crossover pressure area. Finally, the crossover point in the ternary system was between 13 and 15 MPa.

In the ternary system (solute-SC-CO_2_-cosolvent), the enhancement factor has been considered to study the cosolvent effect. This factor is the ratio of the obtained solubility of solute within the ternary system to that of the binary system. By investigating the presented results in Table 3, it can be founded out that the solubility was increased by adding menthol to SC-CO_2_. The cosolvent effect “*e*” was applied to better evaluate the solubility enhancement ^7,64^:

3 $$e=\frac{{y}_{2}^{{^{\prime}} }(P,T,{y}_{3})}{{y}_{2}(P,T)}$$

Table 3 presents the values of "$e"$ in this study. The highest cosolvent effect was (61.2-fold) is related to the pressure of 12 MPa and a temperature of 338 K. Other researchers also reported the cosolvent effect in their studies. Hosseini et al*.*^12^, compared the solubility of clozapine and lamotrigine in SC-CO_2_ (with solid cosolvent (menthol)) with the cosolvent-free condition. The solubility of clozapine showed an approximate 56-fold enhancement while that of lamotrigine was increased almost 8 times. Sabet et al*.*^65^ measured acetaminophen solubility in SC-CO_2_ with and without menthol solid cosolvent under different temperatures and pressures. As shown by the results, menthol strongly augmented acetaminophen solubility by (8.27-fold). Gupta and Thakur^66^ investigated the solubility of phenytoin in SC-CO_2_. They concluded that solid solute solubility in SC-CO_2_ is only 3 µmol/mol while its solubility increased to 1302 µmol/mol (at 45 °C and at 196 bar) in SC-CO_2_ with menthol solid cosolvent. Notably, interactions between menthol and phenytoin resulted in a 400-fold solubility enhancement. Sodeifian and Sajadian^10^ determined the solubility of letrozole under different circumstances in SC-CO_2_ with and without menthol. Solid co-solvent could increase letrozole solubility up to 7.1 folds compared to the binary system (without solid cosolvent).

In general, the increase in the solubility of solids in ternary systems (CO_2_ + cosolvent) can be attributed to the increase in solvent density, dipole–dipole interactions, and also hydrogen bonding between the solute and the cosolvent^67^. In this case, upon adding menthol to the cell, the density of the SCF enhanced, leading to an increment in the solubility. The polarity of SC-CO_2_ can also be affected by the cosolvent. Menthol enhanced the solubility of KTZ in CO_2_ due to the presence of a hydroxyl (polar) group and a hydrocarbon group (nonpolar) in the respective structures. As a result, it can be concluded that stronger attractive polar interaction and hydrogen bonding could lead to greater solubility. Also, by comparing values of *e* in Table 3, it can be inferred that cosolvent effects decreased with the increment of the pressure, which is compatible with the published studies^4–6,11^.

### Correlation of the binary system

**Figure 3:**
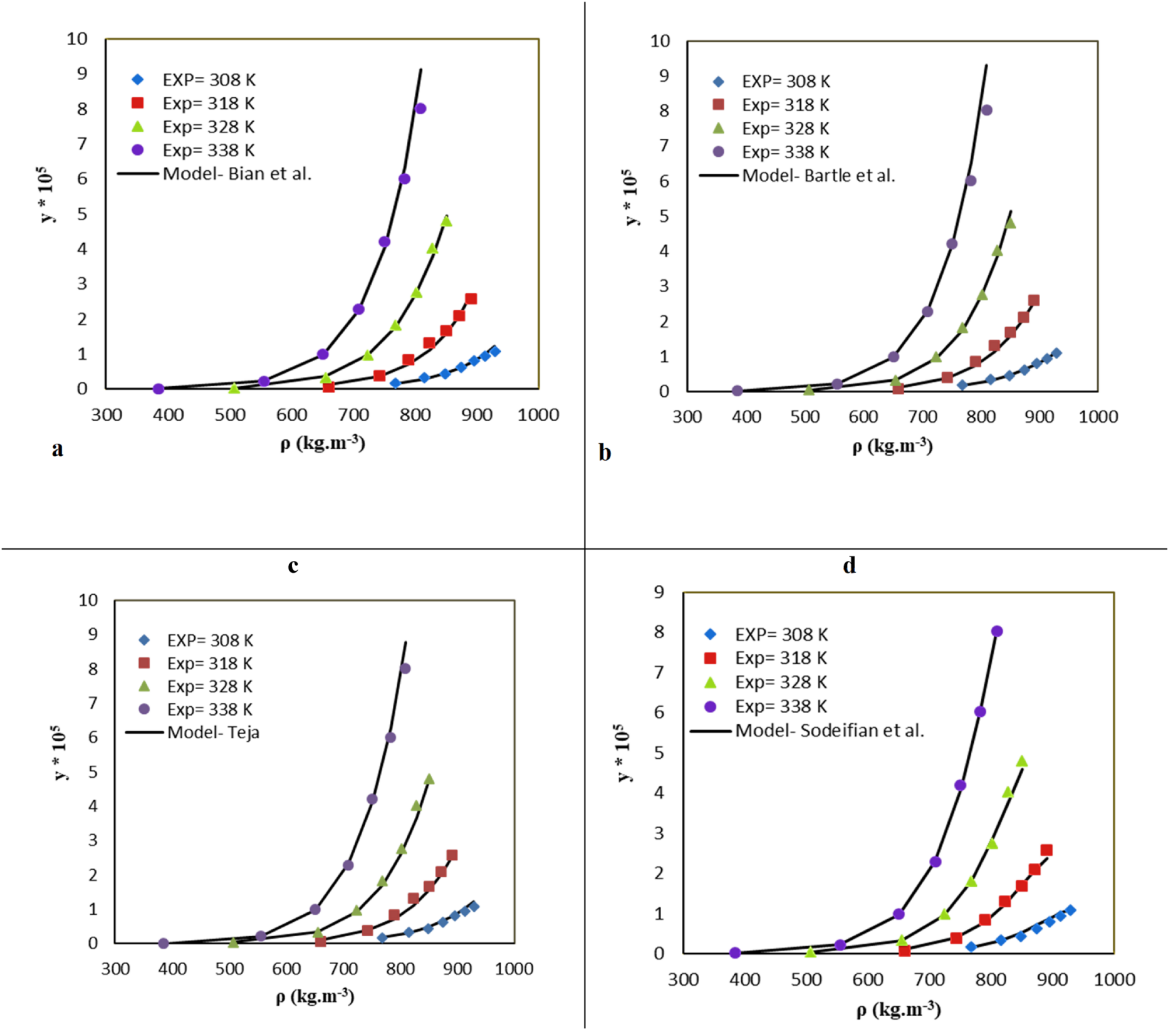
The experimental (points) and computed (line) solubility of KTZ (binary system) by (**a**) Bian et al*.*, (**b**) Bartle et al. (**c**) MST & (**d**) Sodeifian et al*.,* models.

The present study considered ten semiempirical equations for correlating KTZ solubility in SC-CO_2_, as listed in Table 4. Figure 3 depicts the outputs of the correlation at different temperatures. Then, statistical criteria were employed to investigate the abilities of semiempirical models. As a general rule, the more adjustable parameters lead to more accurate correlations. To provide a reliable accuracy criterion to compare the models with different numbers of adjustable parameters, AARD and *R*_*adj*_ with the following equations were used^68^:

4 $$ AARD,\,\% = \frac{100}{{{N_i} - Z}}\sum\limits_{i = 1}^{N_i} {\frac{{\left| {{y_2}^{calc} - {y_2}^{exp}} \right|}}{{{y_2}^{\exp }}}}  $$

**Table 4:** A brief statement of the density-based models utilized in the present research *(c*, *ρ*, *T*, *P*, *P*_*ref*_, *ρ*_*ref*_, *y*_*2*_ and *a*_*0*_*-a*_*5*_ are solubility of solute, density of SC-CO_2_, temperature, pressure, reference pressure, reference density, mole fraction in binary system and adjustable parameters, respectively.

Model	Formula
Chrastil^28^	$c = {\rho^{a_0}}\exp (\frac{{a_1}}{T} + {a_2})$
K-J^29^	$\mathrm{Ln}$(${y}_{2}$) = ${a}_{0}+{a}_{1}\rho +\frac{{a}_{2}}{T}$
Bartle et al*.*^51^	$\ln {\kern 1pt} (\frac{{{y_2}P}}{{{P_{ref}}}}) = {a_0} + \frac{{a_1}}{T} + {a_2}(\rho - {\rho_{ref}})$
MST^32^	$T\ln {\kern 1pt} ({y_2}P) = {a_0} + {a_1}\rho + {a_2}T$
Sparks et al.^42^	$c_2^* = \rho_{r,1}^{{a_0} + {a_1}{\kern 1pt} {\rho_{r,1}}}{\kern 1pt} \exp ({a_2} + \frac{{a_3}}{{T_r}})$
Bian et al.^41^	${y_2} = {\rho^{({a_0} + {a_1}\rho )}}\exp (\frac{{a_2}}{T} + \frac{{{a_3}\rho }}{T} + {a_4})$
Jouyban et al.^33^	$\ln {\kern 1pt} {y_2} = {a_0} + {a_1}\rho + {a_2}{P^2} + {a_3}PT + \frac{{{a_4}T}}{P} + {a_5}\ln (\rho )$
Sodeifian et al.^14^	$\ln {\kern 1pt} {y_2} = {a_0} + {a_1}\frac{{{P^{{\kern 1pt} 2}}}}{T} + {a_2}\ln (\rho T) + {a_3}(\rho \ln \rho ) + {a_4}P\ln T + {a_5}\frac{\ln \rho }{T}$

So that *Z* represents the number of the adjustable variables for each model.

5 $$ {R_{adj}} = \sqrt {\left| {{R^2} - ({{Q(1 - {R^2})} \mathord{\left/ {\vphantom {{Q(1 - {R^2})} {(N - Q - 1))}}} \right. \kern-\nulldelimiterspace} {(N - Q - 1))}}} \right|}  $$

where *N* refers to the numbers of data points in each set. Moreover, *Q* stands for the numbers of the independent variables in each equation. *R*_*adj*_ can be used to compare models with different numbers of independent variables and *R*^2^ represents the correlation coefficient^69^*.*

6 $$ {R^2} = 1 - \frac{{S{S_E}}}{{S{S_T}}} $$

7 $${SS}_{T}=\sum ({{\mathrm{y}}_{\mathrm{exp}})}^{2}-\frac{(\sum {{\mathrm{y}}_{\mathrm{exp}})}^{2}}{\mathrm{N}}$$

8 $${SS}_{E}=\sum {({\mathrm{y}}_{\mathrm{exp}}-{\mathrm{y}}_{\mathrm{model}})}^{2}$$

where *SS*_*E*_ is the error of the sum of squares and *SS*_*T*_ is the total sum of squares.

Correlation results and optimal values of the parameters are presented in Table 5. The mean-values of *AARD%* for Chrastil, Sparks et al., K-J, Bian et al., Bartle et al., MST, Jouyban et al., and Sodeifian et al., models were 10.01, 11.52, 09.93, 09.22, 07.55, 09.61, 15.11 and 06.94%, respectively. According to the ANOVA results, it can be concluded that Bian et al., (*R*_*adj*_ = 0.991), MST (*R*_*adj*_ = 0.996), and Sodeifian et al*.,* (*R*_*adj*_ = 0.999) models could more accurately correlate KTZ solubility (Fig. 3).

**Table 5:** The correlation results of the KTZ – CO_2_ system provided by semi-empirical models (*AARD, R*_*adj*_ and *a*_*0*_*-a*_*5*_ are average absolute relative deviation, adjusted correlation coefficient and adjustable parameters, respectively).

Model	${a}_{0}$	${a}_{1}$	${a}_{2}$	${a}_{3}$	${a}_{4}$	${a}_{5}$	*AARD*%	*R*_*adj*_
Chrastil	11.107	− 11,945.812	− 39.215	–	–	–	10.01	0.990
Sparks et al.,	5.9141	2.3629	25.666	− 41.5872	–	–	11.52	0.989
K-J	16.75	0.0144	− 1273.9	–	–	–	09.93	0.985
Bian et al*.,*	− 4.0301	0.0020	− 10,553.88	− 2.2336	− 10.6230	–	09.22	0.991
Bartle et al*.,*	37.82	− 14,650.1	0.0175	–	–	–	07.55	0.989
MST	− 18,858.47	245,948.22	38.761	–	–	–	09.61	0.996
Jouyban et al.,	− 56.512	− 53.3345	− 0.00002	0.00007	− 1.5936	37.1537	15.11	0.989
Sodeifian et al*.,*	− 25.7435	− 0.503	3.2028	0.0027	0.003600	− 1910.229	06.94	0.999

The energy term describing the temperature term coefficient in Chrastil, Sparks et al*.*, and Bartle et al*.,* models were considered to determine the heat of solvation (ΔH_sol._), the vaporization heat of the solute (ΔH_vap_.), and total heat (ΔH_t_). The second tunable variables of Chrastil, Sparks et al*.,* and Bartle et al*.,* models were used to calculate ΔH_t_ and ΔH_vap_, respectively. Also, ∆H_sol_ was calculated based on the difference between ∆H_vap_ and ∆H_total_. Based on Table 6, the enthalpy of KTZ dissolution in SC-CO_2_ and ∆H_total_ were 99.32 and 101.70 kJ.mol^−1^, respectively. Also, ∆H_vap._ was calculated by Bartle et al., as 121.80 kJ.mol^−1^. According to our data, solvation and vaporization processes are endothermic and exothermic, respectively. The value of ∆H_vap_ was bigger than ∆H_total_. Due to differences between ΔH_total_ and ΔH_vap_ values, ΔH_sol_ values from different models were calculated − 22.48 and − 20.10 kJ.mol^−1^.

**Table 6:** The vaporization (ΔH_vap_), approximated total (ΔH_total_), and solvation (ΔH_sol_) enthalpy for KTZ.

Compound	ΔH_total_ (kJ mol^−1^)	ΔH_vap._ (kJ mol^−1^)^b^	ΔH_sol._ (kJ mol^−1^)^d^
Ketoconazole	99.32^a^	121.80	− 22.48
	105.09^c^	121.80	− 16.71

^a^Obtained from the Chrastil's model.

^b^Obtained from the Bartle et al*.,* model.

^c^Obtained from the Sparks et al*.,* model.

^d^Obtained from the difference between the ΔH_vap_ and ΔH_total_.

### Correlation of the ternary system

**Figure 4:**
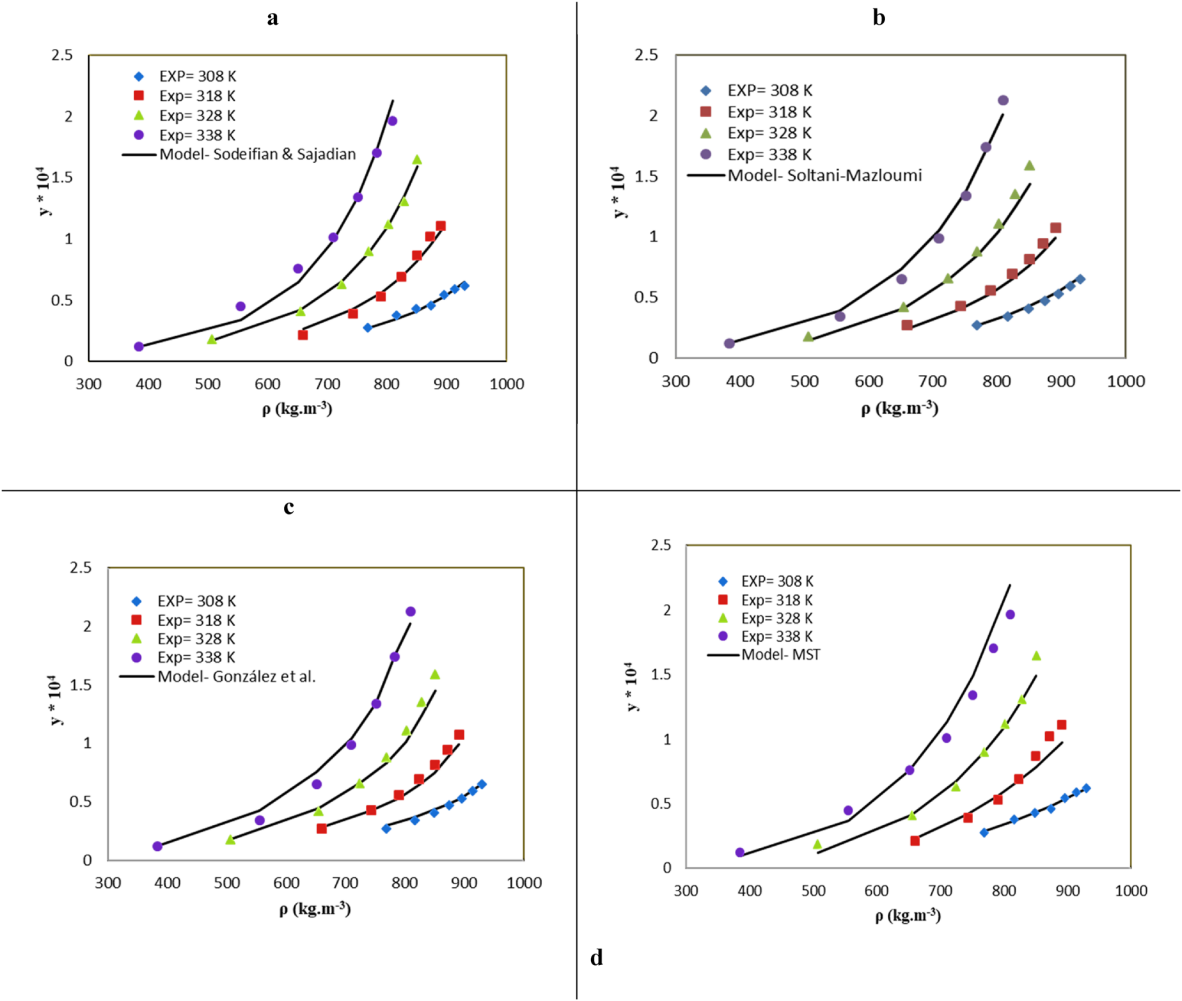
The experimental (points) and calculated (line) of KTZ solubility (ternary system**)** by (**a**) Sodeifian and Sajadian, (**b**) Soltani-Mazloumi, (**c**) González et al*.*, and (**d**) MST models.

The present research assessed the correlation of KTZ solubilities in SC-CO_2_ with menthol by five semiempirical models (Table 7). Menthol solubility in SC-CO_2_ was reported in previous work^58^. The statistical criteria (*i.e., R*_*adj*_ and *AARD*%) were applied to examine the capability of the presented models. A genetic algorithm was also used to obtain adjustable parameters. Figure 4 and Table 8 present the compatibility of KTZ solubility data with the semiempirical results. The highest accuracy was offered by the Sodeifian and Sajadian model (*AARD,%* = *0*6.45, *R*_*adj*_ = 0.995), followed by those of González et al. (*AARD,%* = 07.51, *R*_*adj*_ = 0.991), MST (*AARD,%* = 08.97, *R*_*adj*_ = 0.986) and Soltani and Mazloumi (*AARD,%* = 07.09, *R*_*adj*_ = 0.992), respectively.

**Table 7:** A brief statement of the density-based models utilized in the present research (*ρ*_*1*_, *T*, *P*, *P*_*ref*_, ${y}_{2}^{\mathrm{^{\prime}}}$ , *y*_*3*_ and *a*_*0*_*-a*_*6*_ are density of SC-CO_2_, temperature, pressure, reference pressure, mole fraction in ternary system, mole fraction of cosolvent and adjustable parameters, respectively).

Model	Formula
MST^53^	$T \mathit{ln}\left(\frac{{y}_{2}^{\mathrm{^{\prime}}}P}{{P}_{ref}}\right)={a}_{0}+{a}_{1}{\rho }_{1}+{a}_{2}T+{a}_{3}{y}_{3}$
Sodeifian-Sajadian^58^	$\mathrm{ln}\left({y}_{2}^{\mathrm{^{\prime}}}\right)={(a}_{0}+\frac{{a}_{1}{\rho }_{1}}{T})\mathrm{ln}({\rho }_{1})+{a}_{2}{\rho }_{1}+{a}_{3}\mathrm{ln}({y}_{3}\mathrm{P})$
González et al.^52^	$\mathrm{ln}\left({y}_{2}^{\mathrm{^{\prime}}}\right)={a}_{0}\mathrm{ln}({\rho }_{1})+{a}_{1}\mathrm{ln}({y}_{3})+\frac{{a}_{2}}{T}+{a}_{3}$
Soltani-Mazloumi^49^	$\mathrm{ln}\left({y}_{2}^{\mathrm{^{\prime}}}\right)={a}_{0}+\frac{{a}_{1}}{T}+\frac{{a}_{2}}{T}{\rho }_{1}-{a}_{3}\mathrm{ln}(\mathrm{P})+{a}_{4}\mathrm{ln}({y}_{3}{\rho }_{1}T)$

**Table 8:** The correlation results of the KTZ– Menthol-CO_2_ system provided by the semi empirical models (*AARD, Radj* and *a*_*0*_*-a*_*6*_ are average absolute relative deviation, adjusted correlation coefficient and adjustable parameters, respectively).

Model	${{\varvec{a}}}_{0}$	${{\varvec{a}}}_{1}$	${{\varvec{a}}}_{2}$	${{\varvec{a}}}_{3}$	${{\varvec{a}}}_{4}$	*AARD%*	*R*_*adj*_
MST	− 10,884.4360	3.1002	21.7610	28.803	–	8.97	0.986
González et al*.,*	2.902	0.657	− 4544.27	− 12.26	–	7.51	0.991
Sodeifian—Sajadian	− 2.852	− 1.0502	0.0339	0.153	–	6.45	0.995
Soltani-Mazloumi	10.489	− 8213.5	2.155	0.4203	0.2776	7.09	0.992

## Conclusions

In this research, the KTZ solubility in SC-CO_2_ (with and without menthol) was experimentally measured at the temperature range of 308–338 K and the pressure range of 12–30 MPa using spectrophotometric analysis. The tests were carried out in triplicates to enhance the reliability of the solubility data. Moreover, the mole fractions(*y*) and KTZ solubility (*S* (g/ L)) in SC-CO_2_ (binary system) ranged between 0.001 and 0.784 and 0.2 × 10^–6^ and 8.02 × 10^–5^, while the mole fractions of the drug in the SC-CO_2_ with cosolvent (i.e., the ternary system) ranged in 1.2 × 10^–5^–1.96 × 10^–4^. Therefore, it can be concluded that the solubility increased in the presence of menthol. Several semi-empirical and empirical models were utilized for correlating experimental results of binary and ternary systems. Among them, Sodeifian et al. model managed to correlate the experimental data for the mentioned binary system at higher accuracy. In the case of the ternary system, the highest accuracy was provided by the Sodeifian and Sajadian model.
